# A Matrix-Assisted Laser Desorption Ionization–Time of Flight Mass Spectrometry Direct-from-Urine-Specimen Diagnostic for Gram-Negative Pathogens

**DOI:** 10.1128/spectrum.03730-22

**Published:** 2022-10-18

**Authors:** Hyojik Yang, Richard D. Smith, Kylie P. Sumner, David R. Goodlett, J. Kristie Johnson, Robert K. Ernst

**Affiliations:** a Department of Microbial Pathogenesis, School of Dentistry, University of Maryland, Baltimore, Maryland, USA; b Department of Pathology, School of Medicine, University of Maryland, Baltimore, Maryland, USA; c Department of Biochemistry and Microbiology, University of Victoria, Victoria, British Columbia, Canada; d International Centre for Cancer Vaccine Science, University of Gdansk, Gdansk, Poland; Johns Hopkins Hospital

**Keywords:** lipid A, UTI, Gram-negative bacteria, MALDI-TOF mass spectrometry, fast lipid analysis technique, MALDI-TOF MS, urinary tract infection

## Abstract

Urinary tract infections (UTIs) pose a major public health burden. The vast majority of UTIs are caused by Gram-negative bacteria. Current culture-based pathogen identification methods may require up to 24 to 48 h of incubation. In this study, we developed and evaluated a method for Gram-negative pathogen identification direct from urine, without culture, via matrix-assisted laser desorption ionization–time of flight mass spectrometry (MALDI-TOF MS) in approximately 1 h. Urine samples were collected (*n* = 137) from the University of Maryland Medical Center clinical microbiology laboratory. To identify bacteria direct from urine, two methods were evaluated. First, 1 μL of urine was directly spotted onto the MALDI target plate, and second, 1 mL of urine was centrifuged at 8,000 rpm for 5 min before processing using the fast lipid analysis technique (FLAT). Mass spectra were acquired on the Bruker MALDI Biotyper sirius system in the negative-ion mode. Results were compared to those of standard culture methods. When 1 μL of urine was directly spotted, positive agreement was 81.5% (101/124) and, after centrifugation, 94.4% (117/124) relative to that of standard culture methods. Negative agreement for both methods was 100% (13/13). The time to results for both of the specimen preparation methods using the FLAT extraction protocol was approximately 1 h, with minimal hands-on time required (<5 min). The ability to rapidly identify pathogens directly from urine, without the need for culture, allows for faster turnaround times and, potentially, improved patient outcomes. Overall, the FLAT extraction protocol, in combination with lipid A identification, provides a reproducible and accurate method to rapidly identify urinary pathogens.

**IMPORTANCE** This study describes and evaluates a direct-from-urine extraction method that allows identification of Gram-negative bacteria via MALDI-TOF MS within 1 h. Currently, identification of urinary pathogens requires 24 h of culture prior to identification. While this method may not replace culture, we demonstrate its utility in screening for common urinary pathogens. By providing identifications in under 1 h, clinicians can potentially treat patients sooner with more-targeted antimicrobial therapy. In turn, earlier treatment can improve patient outcome and antimicrobial stewardship. Furthermore, MADLI-TOF MS is a readily available, easy-to-use diagnostic tool in clinical laboratories, making implementation of this method possible.

## INTRODUCTION

Globally, urinary tract infections (UTIs) are one of the most prevalent bacterial infections (1). UTIs pose a substantial health and economic burden, contributing to over one million emergency department visits and 100,000 hospital admissions in the United States annually (2, 3). It is estimated that approximately 50% of people will suffer from a UTI at least once in their lifetime (4, 5). Furthermore, UTIs can lead to potentially life-threatening complications, such as urosepsis (6). Over 80% of UTIs are caused by Gram-negative bacteria, with most infections being caused by Escherichia coli (7).

Current methods for diagnosing UTIs include clinical evaluation of symptoms, urine dipstick, urinalysis, and urine culture (8, 9). In the clinical microbiology laboratory, culture-based methods remain the standard diagnostic method for UTIs, as they allow for pathogen quantification, identification, and, secondarily, antimicrobial susceptibility testing. Typically, a positive UTI is defined by having a bacterial load greater than 100,000 CFU/mL (10). Pathogen identification via culture-based methods requires 24 to 48 h, delaying optimal antimicrobial therapy (11). Thus, identifying pathogens without the need to culture would reduce the time to bacterial identification and targeted antimicrobial therapy, thereby potentially improve patient outcomes.

Recently, matrix-assisted laser desorption ionization–time of flight mass spectrometry (MALDI-TOF MS) has become a critical technology in the clinical microbiology setting. MALDI-TOF MS is a reliable, simple, and readily available technology (12, 13). To date, there are two companies with FDA-approved protein-based MALDI-TOF MS platforms used in clinical microbiology laboratories: MALDI Biotyper sirius (MBT) (Bruker Daltonics, Billerica, MA) and bioMérieux Vitek MS (bioMérieux, Inc., Durham, NC) (12). These platforms analyze highly conserved ribosomal proteins and compare them to a reference database to identify microorganisms (14). Both of these MALDI-TOF MS methods require culture prior to analysis; however, several novel methods have been developed to allow MALDI-TOF MS analysis direct from clinical specimens (8, 15–17).

**FIG 1:**
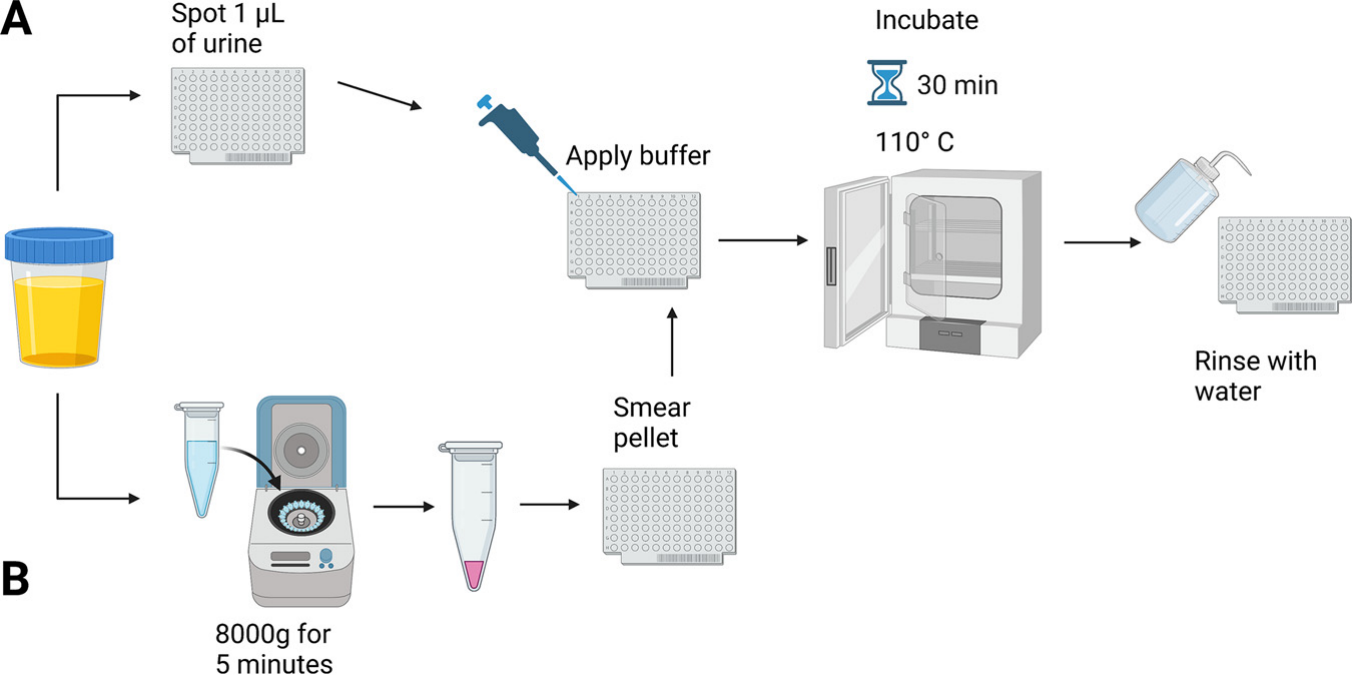
Flow diagram of the FLAT for both methods of specimen processing for direct-from-urine analysis. (A and B) Specimen preparation for FLAT without centrifugation (A) and with centrifugation (B). Both sample preparation methods have a hands-on time of less than 5 min.

Previously, our group has shown the ability to use membrane lipids for the identification of bacterial and fungal pathogens via MALDI-TOF MS (18). To analyze these lipids, a rapid, easy-to-perform extraction method, the fast lipid analysis technique (FLAT) (Fig. 1) was developed. By using the FLAT extraction protocol directly on clinical specimens, pathogens can be identified in less than 1 h with minimal hands-on time (17, 19, 20). Furthermore, the use of the FLAT in combination with lipid MS analysis has demonstrated the ability to identify antimicrobial resistance via MALDI-TOF MS and multiple organisms from a single polymicrobial sample (17–19, 21–23). In this study, we further develop and evaluate the use of the FLAT in combination with lipid A spectra to better identify Gram-negative bacteria, the most prevalent urinary pathogens, directly from clinical urine specimens.

## RESULTS

### Pathogen identification.

A total of 137 urine samples were collected from the University of Maryland Medical Center clinical microbiology laboratory between August 2021 and December 2021, and the pathogens observed included 60 Escherichia coli (48.4%), four Proteus species (3.2%), six Pseudomonas aeruginosa (4.8%), two Citrobacter koseri (1.6%), one Citrobacter freundii (0.8%), 26 Klebsiella pneumoniae (21.0%), two Klebsiella oxytoca (1.6%), three Serratia marcescens (2.4%), three Enterobacter cloacae complex (2.4%), and 17 mixed microbial flora (13.7%) isolates (Table 1). Of the 137 collected urine samples, 13 were negative, with no observed growth by culture.

**TABLE 1:** Identification by lipid-based MALDI-TOF MS after FLAT extraction^*a*^

Pathogen	No. of urine specimens	No. of specimens positive with direct identification/total no.	No. of specimens positive with centrifugation/total no.
Escherichia coli	60	52/60	59/60
Proteus spp.	4	3/4	4/4
Pseudomonas aeruginosa	6	5/6	6/6
Citrobacter koseri	2	0/2	1/2
Citrobacter freundii	1	1/1	1/1
Klebsiella pneumoniae	26	22/26	24/26
Klebsiella oxytoca	2	1/2	1/2
Serratia marcescens	3	1/3	2/3
Enterobacter cloacae complex	3	0/1	2/3
Mixed microbial flora	17	16/17	17/17
			
Total	124	101/124 (81.5%)	117/124 (94.4%)

a Agreement was calculated in reference to culture-based identification.

**FIG 2:**
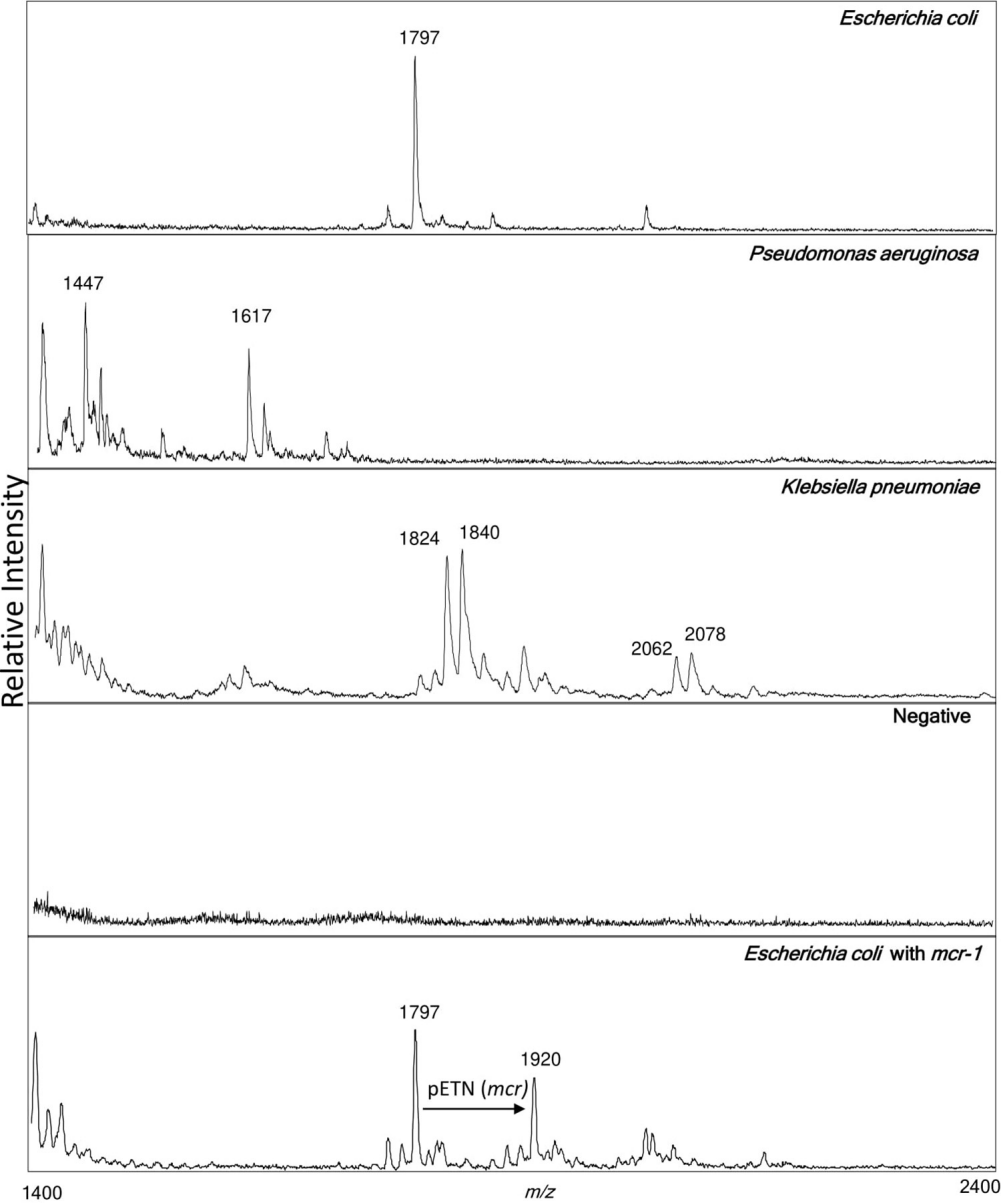
Representative mass spectra from three pathogens, E. coli, Pseudomonas aeruginosa, and Klebsiella pneumonia, direct from an individual urine sample using the FLAT, followed by MS analysis, and a representative mass spectrum from individual urine samples that showed no growth (negative). An example of spectra from an E. coli harboring *mcr-1* is shown at the bottom. All urine samples were previously characterized by the University of Maryland Medical Center clinical microbiology laboratory.

Overall, percent agreements when specimens were processed directly without centrifugation and with centrifugation were 81.5% (101/124) and 94.4% (117/124), respectively (Table 1). Negative agreement for both methods was 100% (13/13). Representative spectra collected by direct-from-urine analysis of specimens containing E. coli, P. aeruginosa, and K. pneumoniae and those with no growth are displayed in Fig. 2.

### Identification of markers for polymyxin resistance.

Three urine samples in this study were observed to have ion species at *m/z* 1,797 and *m/z* 1,920, which is indicative of E. coli with a phosphoethanolamine (PEtN) addition (17, 24) (Fig. 2, E. coli with *mcr-1*). The addition of a PEtN is a known mechanism and marker of polymyxin resistance (25). A PEtN addition is suggestive of an *mcr* gene. The presence of *mcr* genes was confirmed via PCR. All three samples were identified to be harboring *mcr-1.* All three isolates were determined to be colistin resistant by manual broth microdilution via an in-house-made panel.

### Assay reproducibility.

All collected samples were processed separately by two individuals to determine assay reproducibility. For both sample preparation methods, concordance between the two individuals blinded to sample identification and performing the protocol for positive urine specimens was 94.4% (117/124). The discordant spectra between the individuals were typically attributed to low signal-to-noise ratios of the collected spectra. For the 13 no-growth, negative specimens, concordance was 100% (13/13). Overall, the high concordance illustrated the reproducibility of this assay.

## DISCUSSION

The lipid-based MALDI-TOF MS assay using the FLAT extraction protocol has many strengths as a diagnostic platform for UTI diagnosis directly from patient samples. This is a rapid assay that has been shown to be more accurate than previous direct-from-urine MALDI-TOF-based assays, is highly reproducible, and has the potential to identify markers for antibiotic resistance directly from specimens (8, 26–28).

MALDI-TOF MS has revolutionized the field of clinical microbiology due to its fast, reproducible, and cost-effective nature. Furthermore, MALDI-TOF MS diagnostic platforms have extensive pathogen libraries, compared to molecular methods, since they do not require preidentified species-specific primer sets (29, 30). By using the direct-from-specimen FLAT extraction method, the time to identification for Gram-negative bacteria can be reduced to less than 60 min with minimal hands-on time, thus reducing the time to more-targeted antimicrobial therapy and potentially improving overall antimicrobial stewardship and patient outcomes (31).

Previous studies have developed methods to identify urinary pathogens directly from specimens via MALDI-TOF MS (8, 26–28). These previously published methods all utilized the existing protein-based MALDI-TOF platforms, MALDI Biotyper sirius (MBT) or Vitek MS. Overall, these extraction methods had times to results comparable to that of FLAT; however, these methods were much more labor-intensive, requiring multiple centrifugations, wash steps, and reagents. While FLAT does require a unique library and an aqueous-based citric acid buffer that is not commonly used at this time, this assay could be easily implemented in the future. A significant advantage over current protein-based platforms is that the total sample hands-on time is less than 5 min for FLAT, much less than previously published methods.

Use of the FLAT and lipid-based MALDI-TOF MS identification had an overall positive agreement of 81.5% without centrifugation and 94.4% with centrifugation. The improvement in positive agreement after centrifugation was most likely attributed to increasing the concentration of bacteria by creating a bacterial pellet. Previous direct-from-urine assays have demonstrated positive agreement between 79.2% and 91.8% (8, 26–28); however, these studies included Gram-positive bacterial and fungal pathogens. This study included a bias cohort of only Gram-negative bacteria. When positive agreements for Gram-negative bacteria were compared, FLAT yielded better results, with the highest positive agreement among previously published direct-from-urine assays being 93.7% (8, 26–28). Restricting sample collection to Gram-negative bacteria is a limitation of this study; however, most urinary pathogens observed clinically and studied in previous studies were Gram-negative bacteria, with analysis of Gram-positive and fungal pathogens currently ongoing.

As an added benefit, lipid-based MALDI-TOF MS after FLAT extraction has shown the ability to identify antimicrobial resistance directly from urine specimens within 1 h (17, 19, 20). In this study, we further identified the diagnostic signatures that represent the addition of phosphoethanolamine in three of the 60 E. coli isolates from unique individuals (5% of total E. coli isolates expressing the *mcr-1* gene) that will result in resistance to colistin (polymyxin E) via MALDI-TOF MS. The prevalence of E. coli *mcr-1* observed in this study (5%) was surprising, as the reported prevalence of colistin-resistant E. coli is 1.26% globally and 0.5% in the United States (32).

Despite the many strengths of this assay, one minor shortcoming is that this method requires a negative-ion mode. Currently, conventional clinical MALDI-TOF MS instruments such as the Vitek MS (bioMérieux, Durham, NC) and Bruker Microflex LT/SH (Bruker, Billerica, MA) are not equipped with a negative-ion mode. However, FDA-approved MS instruments equipped with a negative-ion mode are rapidly emerging on the market (e.g., Bruker sirius, Shimadzu 8030, and Autobio 2000), allowing for a more rapid uptake of this method (33). Additionally, current spectral identification of organisms is carried out manually, but an automated program that will remove manual interpretation is currently under development. Finally, future development of this assay includes addressing the limited ability to quantitate bacteria via MALDI-TOF MS. The gold standard of diagnosing UTIs and bacteriuria is the quantification of pathogens (6). MS has previously been unable to provide quantitative results, and semiquantitative assay development is limited and inaccurate (34). We are currently developing methods to improve quantification via MALDI-TOF MS directly from urine through the inclusion of synthetically derived lipid molecules as internal standards. In doing so, both pathogen identification and quantification could be conducted without the need to culture and further support the clinical potential of our assay.

Effective diagnostic testing is paramount for improved patient care and appropriate antimicrobial stewardship. Direct-from-specimen diagnostic platforms remove the need to culture, thus substantially reducing the time to pathogen identification. By reducing the time to pathogen identification, the time to effective antimicrobial therapy is potentially reduced, improving patient outcomes. The FLAT extraction method for lipid-based MS has demonstrated the ability to accurately identify pathogens directly from urine samples within 1 h with minimal hands-on time and can identify antimicrobial resistance. Overall, the ability to identify pathogens and resistance markers highlights the strong potential clinical utility of the FLAT extraction protocol, paired with MS, for direct-from-specimen identification of urinary pathogens.

## MATERIALS AND METHODS

### Specimen collection.

Patient urine specimens (*n* = 137) were collected from the University of Maryland Medical Center clinical microbiology laboratory (Baltimore, MD). Positive urine specimens were included in this study if they were determined to have approximately 100,000 CFU/mL or greater. Specimens were collected after urine was cultured on sheep blood and MacConkey agar to ensure that specimens with only Gram-negative bacteria were included in the study. Negative urine specimens were included if no growth was observed after 24 to 48 h of culture at 37°C on solid tryptic soy agar with 5% sheep blood. For this study, cultured specimens that were primarily Gram-positive bacteria or yeast species were excluded. Urine was collected and refrigerated at 4°C in collection containers containing 1% boric acid to create a bacteriostatic environment. All samples were processed within 5 days of collection. This study was approved by the University of Maryland Institutional Review Board (HP-00064919).

### Sample processing for MALDI-TOF MS analysis.

Urine samples were processed via two methods (Fig. 1). First, 1 μL of urine was spotted directly onto the MALDI target plate. Second, 1 mL of urine was centrifuged at 8,000 × g for 5 min to form a bacterial pellet; then, the bacterial pellet was smeared onto a MALDI target plate. For this study, a stainless-steel, 96-well, disposable Hudson Surface Technology MALDI target plate was used. After the sample was applied to the MALDI target plate, lipids were extracted for analysis using FLAT by the previously published protocol (20). Briefly, to conduct FLAT, 1 μL of buffer containing 0.2 M anhydrous citric acid, pH 4.5, and 0.1 M trisodium citrate dihydrate was placed on top of the sample. Next, the MALDI target plate was incubated at 110°C for 30 min in a humidified chamber and rinsed by gently pipetting 500 μL of endotoxin-free water over the target plate. Each sample was spotted onto the MALDI plate in duplicate. Subsequently, 1 μL of a 10-mg/mL concentration of norharmane matrix suspended in 2:1 chloroform-methanol was spotted onto the extracted lipid sample on a MALDI target.

### MALDI-TOF MS analysis and pathogen identification.

MALDI-TOF MS analysis was conducted using a MALDI Biotyper sirius system (MBT) in the negative-ion linear mode. Analysis was conducted at 50% global intensity with approximately 300 laser shots for each acquired spectrum. Spectra were collected between *m/z* 1,000 and *m/z* 2,400. An electrospray tuning mix (Agilent, Palo Alto, CA) was used for mass calibration. Flex analysis (v3.4) software processed the mass spectra with smoothed and baseline corrections.

After MALDI-TOF MS analysis, pathogens were identified by comparison of sample spectra to previously developed reference spectra by an individual blinded to the identity of each sample spectrum (18). The reference spectral library was a collection of previously acquired spectra from isolates processed for previous studies using the FLAT extraction protocol (18–23). Urine specimens were considered negative if no signature ions were identified with a signal-to-noise ratio (SNR) greater than 3. Positive agreement of lipid-based identification via MALDI-TOF MS for both sample preparation methods was calculated in comparison to culture-based MALDI-TOF identification performed by the clinical microbiology laboratory Vitek MS (bioMérieux, Durham, NC). Negative agreement was calculated using the proportion of no-growth specimens that had spectra determined to have negative identifications.

The cohort included specimens determined to be “mixed microbial flora” by traditional culture methods. “Mixed microbial flora” is used as a designation if multiple, nonpredominating organisms are seen. Typically, for “mixed microbial flora” cultures, individual pathogen identifications are not provided in the chart; thus, identifications were considered accurate if multiple pathogen identifications of any type were observed (35). All other positive urine specimens had a single Gram-negative pathogen.

### PCR to confirm presence of *mcr* genes.

Upon MS analysis, three clinical urine samples from independent patients were identified to have an addition of a phosphoethanolamine (PEtN), a known mechanism of polymyxin resistance (36). The presence of a PEtN was indicative of clinical specimens that harbored a mobilized colistin resistance (*mcr-1*) gene (37). For confirmation, the three suspected specimens were screened via PCR for *mcr-1* to *mcr-5* by using primers previously published by Roschanski et al. (38). The following cycling conditions were executed for the PCR: denaturation for 5 min at 94°C, followed by 25 cycles of amplification at 94^º^C for 30 s and 58°C for 90 s, and then a final extension of 72°C for 60 s (37, 38). Results of the PCR were analyzed via agarose gel electrophoresis.

### Evaluation of assay reproducibility.

To evaluate the reproducibility of the assay, two individuals blinded to sample identification processed the clinical specimens in parallel. Spectra and subsequent identifications, via base peaks acquired from both individuals for each collected sample specimen was compared visually to analyze agreement between the two individuals. Reproducibility was calculated as the proportion of spectra that were concordant between the two blinded individuals out of the total samples collected.
